# Triptolide prevents bone loss via suppressing osteoclastogenesis through inhibiting PI3K‐AKT‐NFATc1 pathway

**DOI:** 10.1111/jcmm.15229

**Published:** 2020-04-28

**Authors:** Jin Cui, Xiaoqun Li, Sicheng Wang, Yiming Su, Xiao Chen, Liehu Cao, Xin Zhi, Zili Qiu, Yao Wang, Hao Jiang, Biaotong Huang, Fang Ji, Jiacan Su

**Affiliations:** ^1^ Department of Orthopedics Shanghai Changhai Hospital Second Military Medical University Shanghai China; ^2^ China‐South Korea Bioengineering Center Shanghai China; ^3^ Graduate Management Unit Shanghai Changhai Hospital Second Military Medical University Shanghai China; ^4^ Zhongye Hospital Shanghai China; ^5^ Institute of Translational Medicine Shanghai University Shanghai China; ^6^ Jinling High School Nanjing China

**Keywords:** bone loss, ovariectomy, PI3K‐AKT‐NFATc1, triptolide, tumour

## Abstract

Bone loss (osteopenia) is a common complication in human solid tumour. In addition, after surgical treatment of gynaecological tumour, osteoporosis often occurs due to the withdrawal of oestrogen. The major characteristic of osteoporosis is the low bone mass with micro‐architectural deteriorated bone tissue. And the main cause is the overactivation of osteoclastogenesis, which is one of the most important therapeutic targets. Inflammation could induce the interaction of RANKL/RANK, which is the promoter of osteoclastogenesis. Triptolide is derived from the traditional Chinese herb *lei gong teng*, presented multiple biological effects, including anti‐cancer, anti‐inflammation and immunosuppression. We hypothesized that triptolide could inhibits osteoclastogenesis by suppressing inflammation activation. In this study, we confirmed that triptolide could suppress RANKL‐induced osteoclastogenesis in bone marrow mononuclear cells (BMMCs) and RAW264.7 cells and inhibited the osteoclast bone resorption functions. PI3K‐AKT‐NFATc1 pathway is one of the most important downstream pathways of RANKL‐induced osteogenesis. The experiments in vitro indicated that triptolide suppresses the activation of PI3K‐AKT‐NFATc1 pathway and the target point located at the upstream of AKT because both NFATc1 overexpression and AKT phosphorylation could ameliorate the triptolide suppression effects. The expression of MDM2 was elevated, which demonstrated the MDM‐p53‐induced cell death might contribute to the osteoclastogenesis suppression. Ovariectomy‐induced bone loss and inflammation activation were also found to be ameliorated in the experiments in vivo. In summary, the new effect of anti‐cancer drug triptolide was demonstrated to be anti‐osteoclastogenesis, and we demonstrated triptolide might be a promising therapy for bone loss caused by tumour.

## 1 INTRODUCTION

In human solid tumour, Bone loss (osteopenia) is one of the most common complications.^1^, ^2^ Surgical treatment is an effective therapy for the treatment of gynaecological tumour, such as cervical cancer, ovarian cancer and endometrial cancer.^3^, ^4^, ^5^ However, after complete hysterectomy and bilateral ovariectomy (OVX), osteoporosis often occurs due to the oestrogen deficiency.^6^, ^7^ Osteoporosis is characterized by decreased bone density and strength due to excessive loss of bone protein and mineral content.^8^ The specific pathogenesis mechanism of osteoporosis is still not completely clarified. However, it has been demonstrated by multiple researches that the imbalance between bone formation and bone resorption is the major cause.^9^ During the pathogenesis of osteoporosis, inflammation‐induced osteoclastogenesis overactivation after menopausal contributed most to the bone metabolism imbalance.^10^, ^11^ Thus, one of the important strategies to prevent and treat osteoporosis is to suppress inflammation.^12^, ^13^, ^14^ The origination of osteoclastogenesis is RANK/RANKL interaction. After activated by RANKL, RANK recruits receptor‐associated factor 6 (TRAF6) and other TRAFs.^15^ Following, multiple downstream signalling pathways, including NF‐κB, AKT and MAPKs, are activated. Through these pathways, bone resorption is induced by the transcription and expression of osteoclast‐specific genes, such as tartrate‐resistant acid phosphatase (TRAP), cathepsin K, matrix metalloproteinase 9 (MMP‐9) and nuclear factor of activated T cells, c1 (NFATc1).

Recently, several researches have demonstrated their new finding of anti‐osteoporosis medicine from traditional Chinese herbs.^16^ Triptolide is isolated from the traditional Chinese herb *lei gong teng* (thunder god vine, *Tripterygium wilfordii Hook.f*). It has been reported that triptolide could suppress cell growth and induce apoptosis in multi‐range of cancer cells in human,^17^, ^18^ and presented anti‐inflammation functions.^19^ Thus, we hypothesized that triptolide could suppress the proliferation and bone resorption ability of osteoclast, which could be a promising therapy for the treatment of bone loss caused by tumour.

## 2 MATERIALS AND METHODS

### 2.1 Reagents and antibodies

**FIGURE 1:**
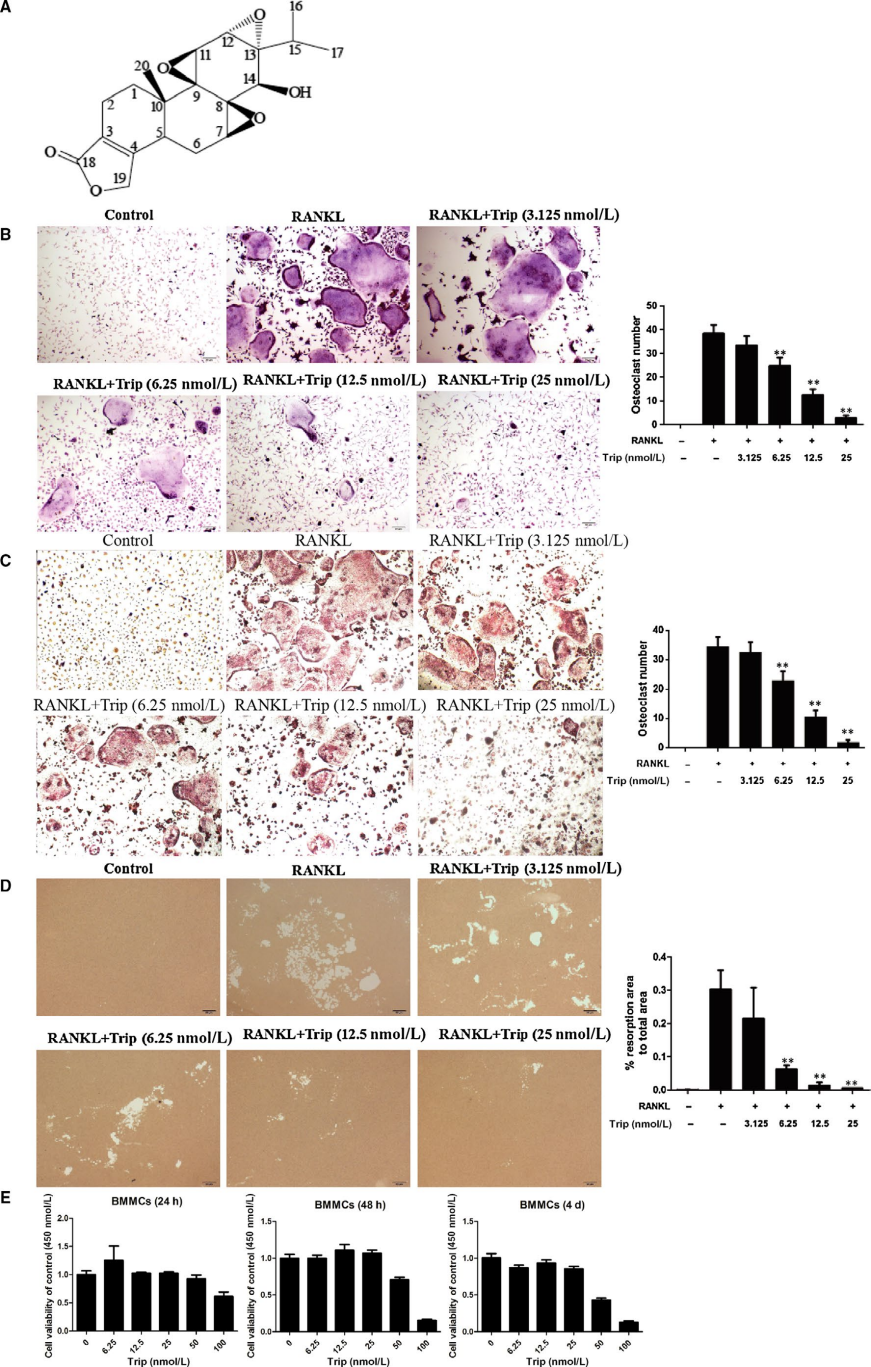
Triptolide inhibits RANKL‐induced osteoclast differentiation and bone resorption in vitro. A, Chemical structure of triptolide. B, Formation of tartrate‐resistant acid phosphatase (TRAP)‐positive cells from RAW264.7 cells and the quantification of formed osteoclasts. C, Formation of TRAP‐positive cells from BMMCs and the quantification of formed osteoclasts. D, The resorption area on the bone biomimetic synthetic surface was quantified by image analysis. E, The CCK‐8 analysis of triptolide cytotoxicity in BMSCs (*P < .05, **P < .01, ***P < .001)

Triptolide (Figure 1A) and dimethyl sulfoxide (DMSO) were purchased from Puhe Biotechnology Co. Prof. J. Hou (Second Military Medical University) supplied the RAW 264.7 cells. Penicillin, streptomycin and foetal bovine serum were also obtained from Puhe Biotechnology Co. SC79 purchased from Abcam.

### 2.2 Cell viability assay

To investigate the cell viability of triptolide, the CCK‐8 assay was carried out according to the manufacturer's instructions. BMMCs were cultured in 96‐well plates at densities of 4 × 10^4^ cells/well without or with triptolide (6.25, 12.5, 25.0, 50.0 or 100.0 nM) for 24 hours, 48 hours and 4 days. After co‐cultural, 10 µL CCK‐8 was added into the plate. After 4‐hours incubation, the absorbance was measured at a wavelength of 450 nm using a microplate spectrophotometer.

### 2.3 In vitro osteoclastogenesis assay

Bone marrow mononuclear cells (BMMCs) were collected from 8‐week‐old C57BL/6 mice,^20^ which were purchased from Slack. Before the experiments, the mice were kept in a specific‐pathogen‐free (SPF) animal laboratory of our hospital (certification: SCXK (Shanghai) 2007‐0003). FBS, DMEM low glucose medium and the mixture of penicillin‐streptomycin were used for the BMMCs culture to enough number. Following, the BMMCs were induced to osteoclasts with RANKL (50 ng/mL) and M‐CSF (20 ng/mL), without or with triptolide (3.125, 6.25, 12.5 or 25.0 nM) for 7 days. According to the manufacturer's instructions, osteoclasts were detected with TRAP staining. RAW264.7 cells were also induced to osteoclasts with RANKL (50 ng/mL) and M‐CSF (20 ng/mL), without or with triptolide (3.125, 6.25, 12.5, or 25.0 nM) for 7 days before osteoclasts detection with TRAP staining. Multi‐nucleated (BMMCs nuclei > 5 or RAW264.7 nuclei > 5) TRAP^+^ cells were counted as osteoclasts.^21^, ^22^

For further investigation of the role of triptolide in the suppression of PI3K‐AKT‐NFATc1, SC79 (the AKT activator) and the overexpression plasmid of NFATc1 were also used during the cell culture.

### 2.4 In vitro bone resorption pit assay

Bone resorption effects of osteoclasts were detected with a hydroxyapatite‐coated plate (Corning).^23^ RAW264.7 cells were seeded in the plate with the concentration of 1 × 10^4^ cells/well and induced to osteoclasts with 50 ng/mL RANKL, without or with different concentrations of triptolide (3.125, 6.25, 12.5 or 25.0 nM). Medium was changed every 3 days. After 7 days, 10% bleach solution was used to clean the plate. After air‐dried under room temperature for 3‐5 hours, the bottom of plate was pictured with light microscope (OLYMPUS, IX71) to record the pits information. The Image‐Pro Plus 6.0 software was used for pits area quantification.

### 2.5 Western blot analysis

Immunoblot analysis was used for key protein quantification. CTR, MMP‐9, Cathepsin K, TRAP and NFATc1 as osteoclastogenesis‐related markers were detected for osteoclastogenesis evaluation. Key protein in PI3K‐AKT‐NFATc1 pathway and the phosphorylation were also detected. MDM2 expression was detected for mechanism investigation.

Total protein was extracted with M‐PER mammalian protein extraction reagent (Pierce). Equal amounts of protein (10 μg per lane) estimated by a bicinchoninic acid protein assay kit (Pierce) were loaded onto (11%) SDS‐PAGE gels and transferred onto nitrocellulose membranes. The used primary antibody in this experiment included mouse anti‐Cathepsin K (1:500), anti‐CTR (1:200), anti‐MMP‐9 (1:400), anti‐NFATc1 (1:250), anti‐TRAF6 (1:350), anti‐TRAP (1:350), anti‐MDM2 (1:150), anti‐AKT (1:250), anti‐P‐AKT (1:400), anti‐PI3K (1:250), anti‐P‐PI3K (1:400) and anti‐beta‐actin (1:1000) (Santa Cruz). HRP‐conjugated anti‐mouse/rabbit antibody (Santa Cruz) was used for the secondary antibody. The final results were detected by chemiluminescence.

### 2.6 Animal preparation

All procedures related to animal experiments of this study were approved by the standards of the Ethics Committee of Second Military Medical University and conformed to the US National Institutes of Health guidelines for use of animals in research. Female C57BL/6 mice, 8‐week‐old, were obtained from Slack, and the SPF animal laboratory (certification: SCXK (Shanghai) 2007‐0003) of Changhai Central Laboratory were used for mice implementing before experiments.

### 2.7 Ovariectomy animal model

The mice were randomly assigned to four groups including the following: sham group, ovariectomized (Model) group, ovariectomized mice treated with triptolide (Trip group), or treated with DMSO (DMSO group). Each group consists six mice. The OVX surgery were carried out as described in our former study.^24^ The mice in Trip group were intraperitoneally (i.p.) given the triptolide (66.7 µg/kg), which was dissolved in DMSO. And the mice in DMSO group were injected the same dose of DMSO. The treatment procedure lasted for 6 weeks. After the treatment, mice were executed with chloral hydrate. Serum was collected for biochemistry examination, and bilateral femurs were excised and fixed in 4% paraformaldehyde solution for histologic and micro‐CT analysis.

### 2.8 Histomorphometric examination

After the 4‐day fixation of right femurs, it took 2 weeks to decalcify the femurs with 10% tetracycline‐EDTA aqueous solution. Then, haematoxylin and eosin (H&E) staining and TRAP staining were carried out after the femurs were paraffin‐embedded to prepare 4‐mm sections. H&E‐stained sections were used for observing the bone trabecula and TRAP‐stained sections were used for osteoclasts observation. The region of the metaphysis was selected to count the number of osteoclasts. The count procedure was done with the software Image‐Pro Plus 6.0.

### 2.9 Micro‐CT analysis

A 9‐μm resolution micro‐CT instrument was used to scan the left femurs. A volume of slices spanning a 1‐mm distance, starting 0.5 mm from the bottom of the growth plate was selected to analyse trabecular bone. Three‐dimensional and two‐dimensional bone structure image slices were reconstructed with the build‐in software of micro‐CT. Total bone mineral density (BMD), bone volume fraction (BV/TV), trabecular number (Tb. N), trabecular pattern factor (Tb. Pf) and bone surface area expressed per unit total volume (BS/TV) were measured.

### 2.10 Serum biochemistry

Blood was collected from the left ventricular, followed with centrifugation process of 1000 *g*  for 5min. ELISA kits were used to determine the serum concentration of bone generation biomarker osteocalcin (OCN), and bone resorption biomarkers IL‐6, TNF‐α and TRAcp5B. The ELISA procedure was done according to the manufacturer's instructions of the ELISA kits (Anogen).

### 2.11 Statistical analysis

IBM SPSS Statistics 22.0 was used to perform all the statistical analysis. The results are presented as mean ± SD. The Student's *t* test was done to compare differences between two groups, and the one‐way ANOVA was done for the differences among more than two groups. If *P* < .05, the differences were regarded as statistically significant.

## 3 RESULTS

### 3.1 Triptolide inhibits RANKL‐induced osteoclastogenesis and bone resorption in vitro

Different concentrations triptolide under the vital dose were used to treat BMMCs and RAW264.7 cells to investigate the effect on osteoclastogenesis. The TRAP staining results of BMMCs are shown in Figure 1B, and the TRAP staining results of RAW264.7 cells are shown in Figure 1C. The numbers of osteoclast were also counted and shown with columns. The results indicated that RANKL treatment significantly increased the number of osteoclasts, and triptolide could significantly ameliorate the increase in dose‐dependent manner. Bone resorption pits were shown in Figure 1D, which showed that triptolide significantly inhibited the bone resorption function of osteoclasts in a dose‐dependent manner. The CCK‐8 experiments indicated that triptolide was not cytotoxic below 25 nM in BMMCs and osteoclasts (Figure 1E and Figure S1).

### 3.2 Triptolide inhibits expressions of osteoclastogenesis‐related markers

**FIGURE 2:**
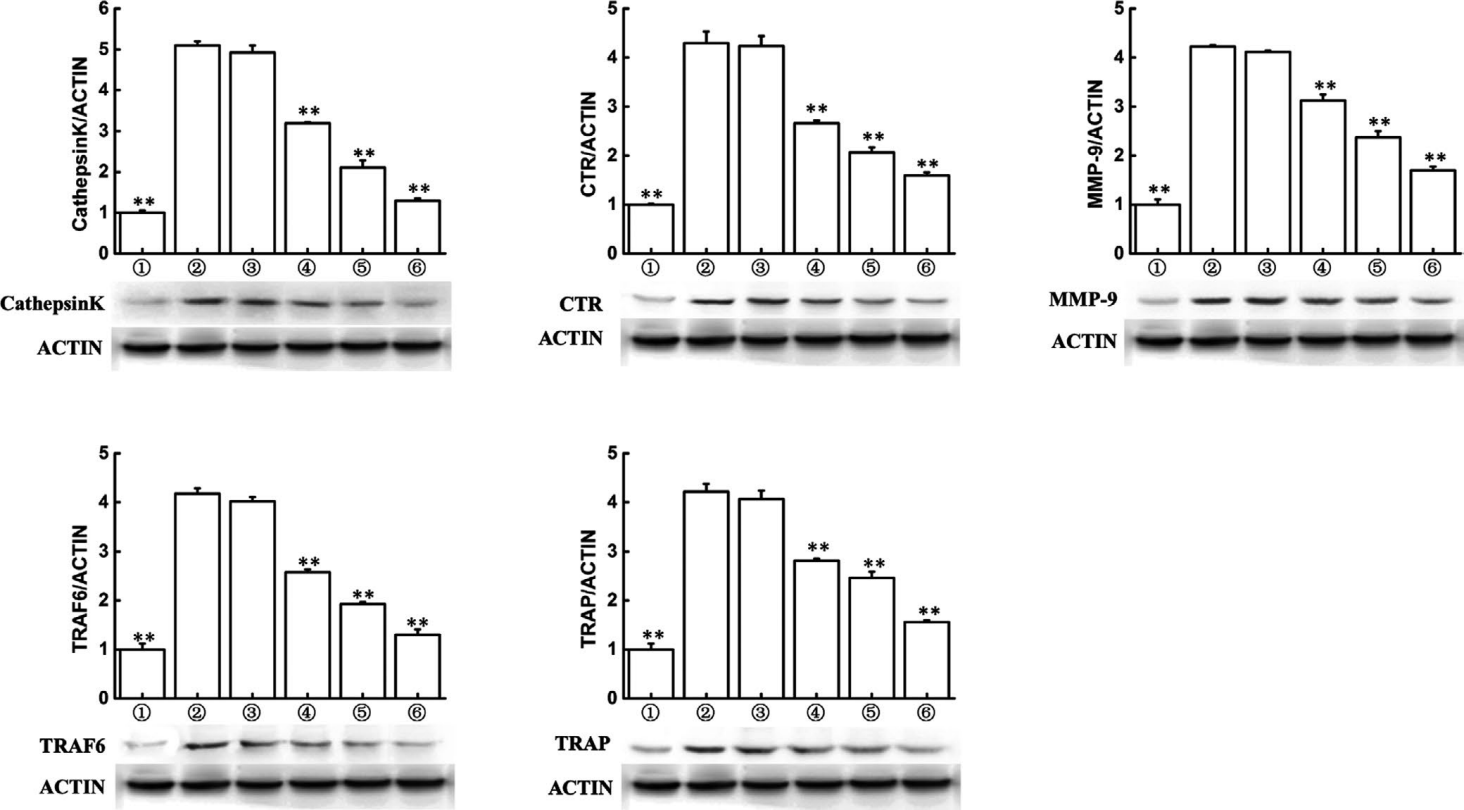
Triptolide inhibits expressions of osteoclastogenesis‐related markers. Western blot and optical density analysis of expression of Cathepsin K, CTR, MMP‐9, TRAF6 and tartrate‐resistant acid phosphatase with beta‐actin as reference (**P < .01, *P < .05 vs RANKL‐induced group)

The osteoclastogenesis was reflected by expressions of several biomarkers, including Cathepsin K, CTR, MMP‐9, TRAF6 and TRAP. The results are shown in Figure 2A‐E, which indicated that RANKL treatment significantly enhanced the expression of these biomarkers (*P* < .01), and triptolide (>6.25 nM) inhibited the RANKL‐induced expression (*P* < .01).

### 3.3 Triptolide suppresses PI3K‐AKT‐NFATc1 pathway activation

**FIGURE 3:**
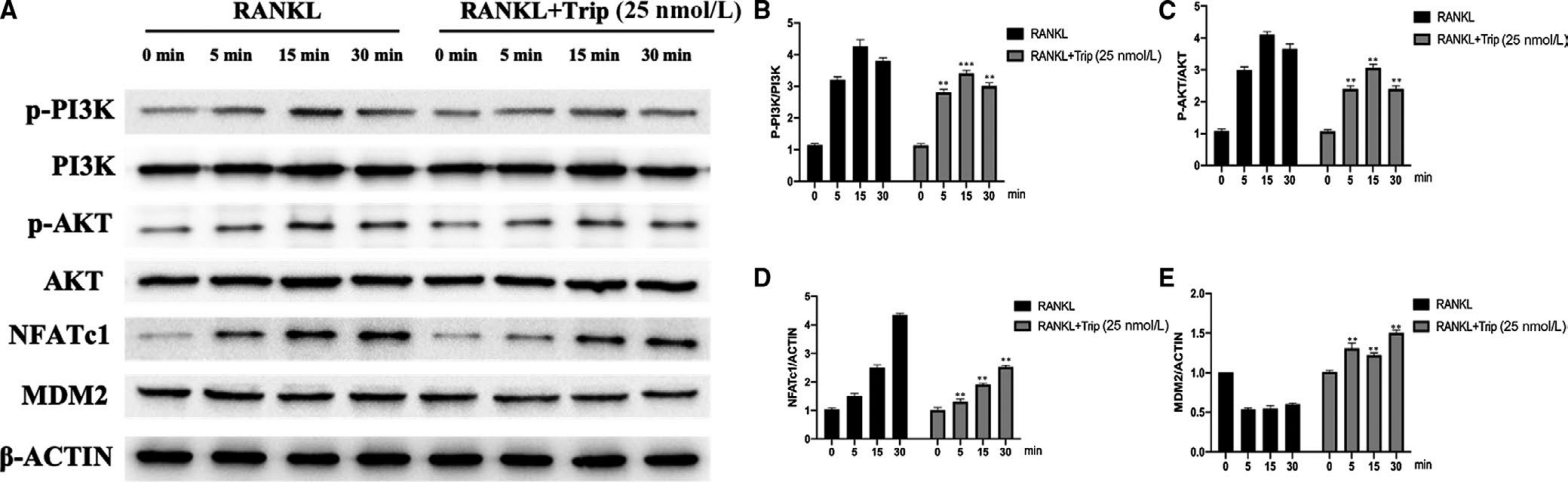
Triptolide suppresses PI3K‐AKT‐NFATc1 pathway activation and promotes MDM2 expression. A, Western blot results of phosphorylation of key protein in PI3K‐AKT‐NFATc1 pathway and MDM2 protein expression. B‐E, Quantification of Western blot results of PI3K, AKT, NFATc1 and MDM2 (*P < .05, **P < .01 vs 0 min group)

Key proteins and the phosphorylation were detected by Western blot assays, and the results are shown in Figure 3A‐D. As the results indicated, the phosphorylation of PI3K, AKT, and the expression of NFATc1 were significantly enhanced by RANKL treatment (*P* < .01), and the effect showed time‐dependent manner. However, the RANKL‐induced activation could be significantly inhibited with triptolide (25 nM) at the same time‐point.

### 3.4 Triptolide promotes MDM2 expression in RANKL‐induced osteoclast in vitro

According to previous study,^25^ PI3K‐AKT pathway could regulate cell cycle through regulating MDM translocation. MDM2 was also detected with Western blot assay, and the results were shown in Figure 3A,E. As the results indicated the expression of MDM2 was decreased after treated with RANKL and enhanced with triptolide treatment.

### 3.5 NFATc1 overexpression reverses triptolide effects on osteoclastogenesis

**FIGURE 4:**
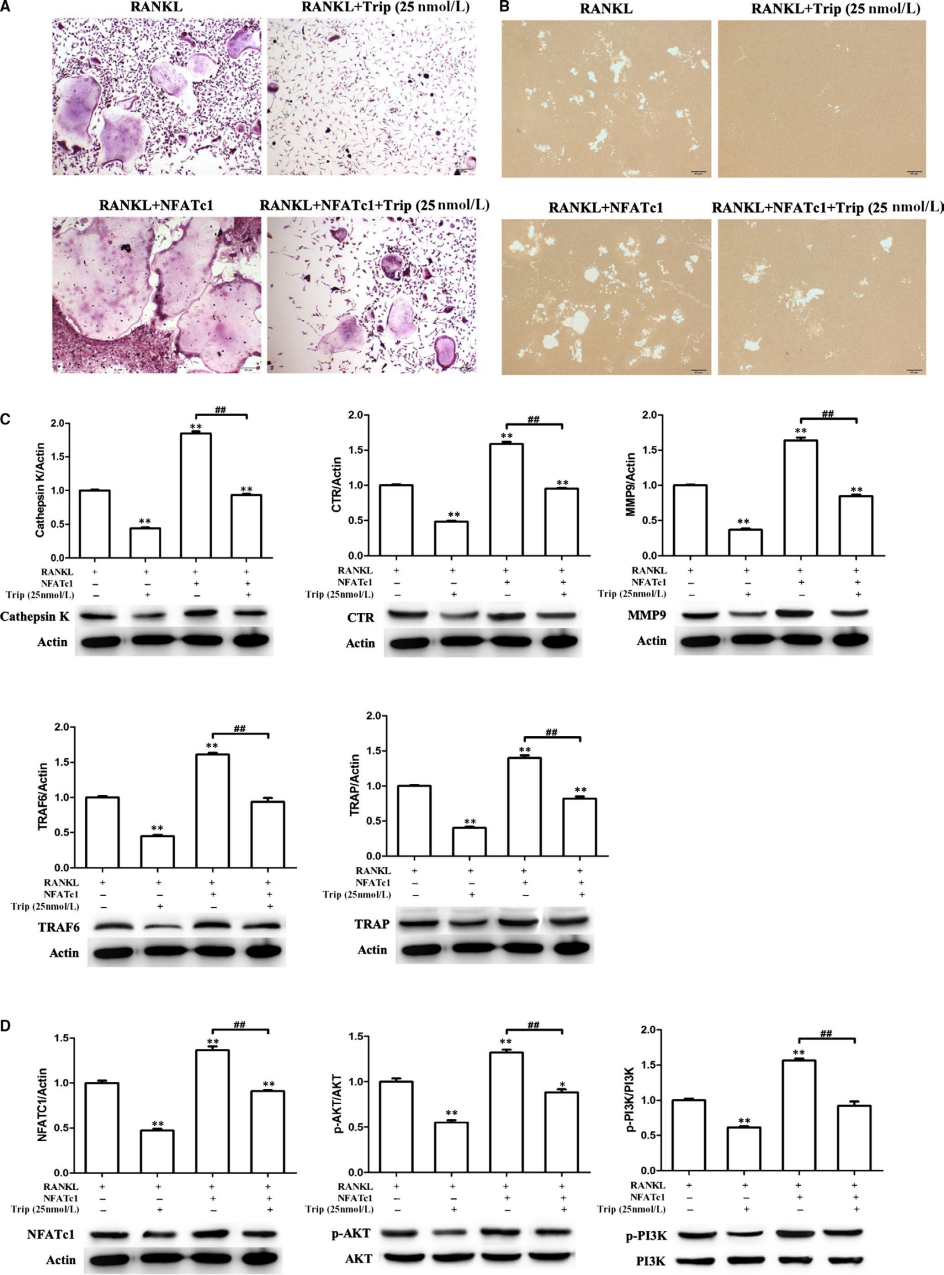
NFATc1 overexpression partially ameliorates the effect of triptolide. A, Formation of tartrate‐resistant acid phosphatase (TRAP)‐positive cells from RAW264.7 cells. B, The resorption area on the bone biomimetic synthetic surface. C, Western blot detected expressions of osteoclastogenesis‐related markers. Western blot and optical density analysis of expression of Cathepsin K, CTR, MMP‐9, TRAF6 and TRAP with beta‐actin as reference. D, Expression of phosphorylation of AKT, PI3K and NFATc1 detected by Western blot assay (**P < .01, *P < .05 vs RANKL‐induced group; ##*P* < .01, #*P* < .05)

To further investigate the mechanism of triptolide suppressing PI3K‐AKT‐NFATc1 pathway in osteoclastogenesis, NFATc1 overexpression plasmid was used to treat RAW264.7 with M‐CSF, RANKL and triptolide (25 nM). As TRAP staining results shown in Figure 4A, triptolide significantly decreased the RANKL‐induced osteoclasts, however, NFATc1 plasmid reverses the effect. As shown in Figure 4B, there were similar results of bone resorption experiment, NFATc1 plasmid reversed the inhibition effect of triptolide on RANKL‐induced osteoclasts function. NFATc1 plasmid also reversed the inhibition effects of triptolide on osteoclastogenesis‐related biomarkers and PI3K‐AKT‐NFATc1 pathway activation (Figure 4C,D). The reverse effect of NFATc1 overexpression indicated that triptolide targeted to the upstream of NFATc1.

### 3.6 AKT agonist reverses triptolide effects on osteoclastogenesis

**FIGURE 5:**
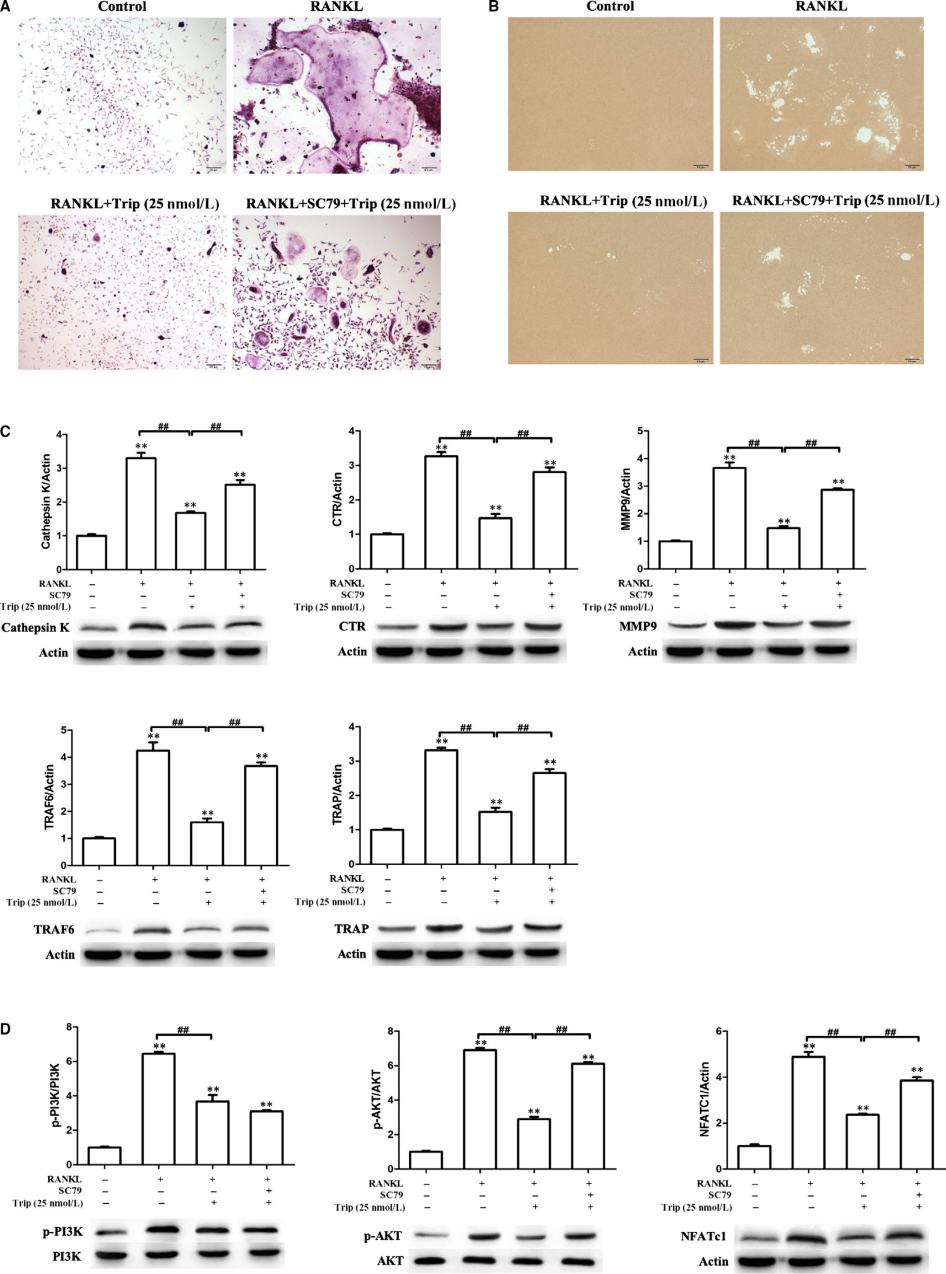
SC79 partially ameliorate the effect of triptolide. A, Formation of tartrate‐resistant acid phosphatase (TRAP)‐positive cells from RAW264.7 cells. B, The resorption area on the bone biomimetic synthetic surface. C, Western blot detected expressions of osteoclastogenesis‐related markers. Western blot and optical density analysis of expression of Cathepsin K, CTR, MMP‐9, TRAF6 and TRAP with beta‐actin as reference. D, Expression of phosphorylation of AKT, PI3K and NFATc1 detected by Western blot assay (**P < .01, *P < .05 vs RANKL‐induced group; ##*P* < .01, #*P* < .05)

The AKT phosphorylation agonist SC79 was also used to investigate the mechanism of triptolide effects on osteoclastogenesis. SC79 (5 μg/mL) was used to treat RAW264.7 with M‐CSF, RANKL and triptolide (25 nM). As TRAP staining results showed in Figure 5A, triptolide significantly decreased the RANKL‐induced osteoclasts, however, SC79 reverses the effect. As shown in Figure 5B, there were similar results of bone resorption experiment, SC79 reversed the inhibition effect of triptolide on RANKL‐induced osteoclasts function. SC79 also reversed the inhibition effects of triptolide on osteoclastogenesis‐related biomarkers and PI3K‐AKT‐NFATc1 pathway activation (Figure 5C,D). The reverse effect of SC79 indicated that triptolide targeted to the upstream of AKT.

### 3.7 Triptolide inhibits ovariectomy‐induced bone loss in vivo

**FIGURE 6:**
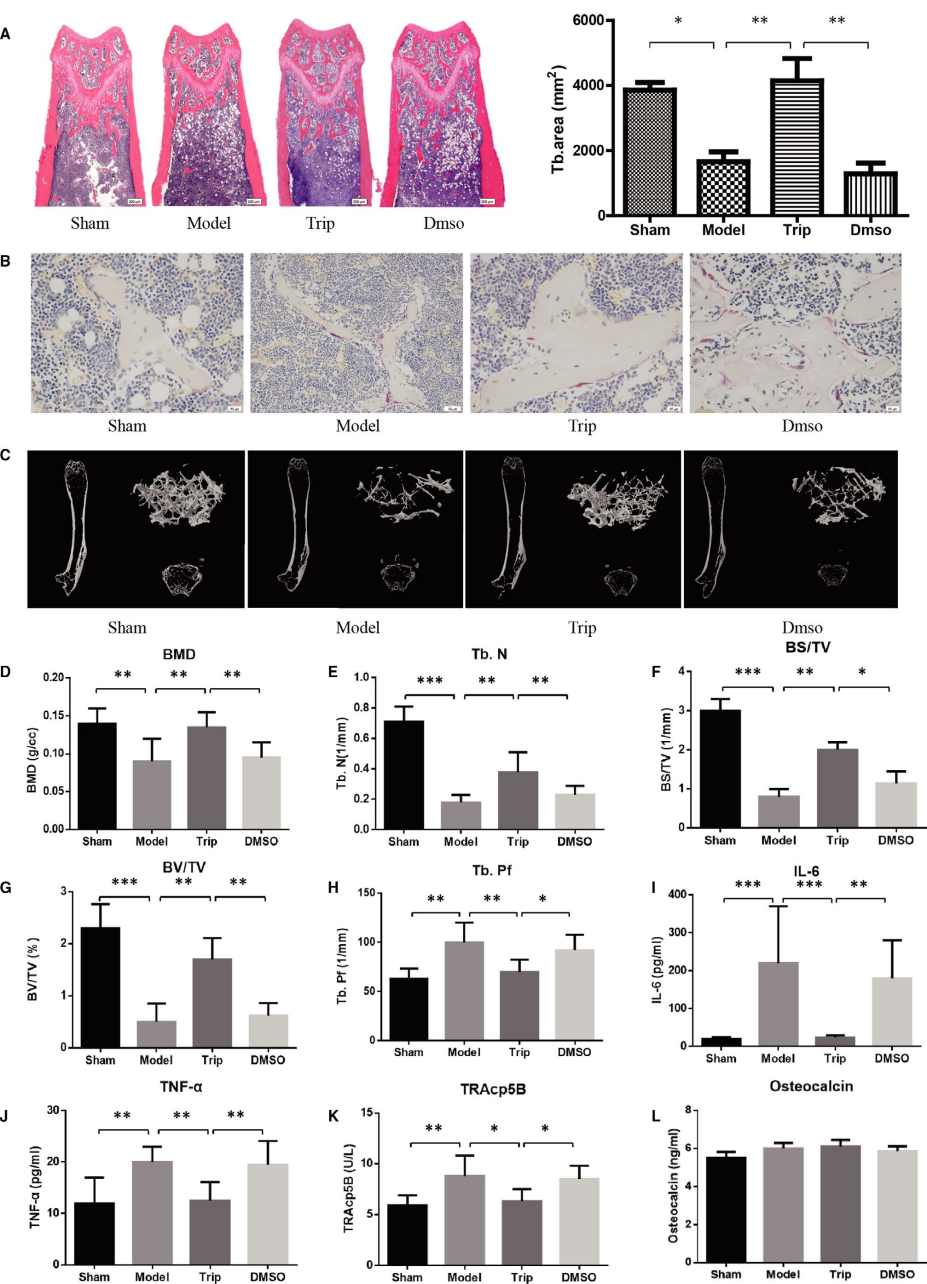
Triptolide prevents ovariectomy‐induced bone loss in vivo. A, Representative H&E staining of distal femoral sections and quantification of trabecular area from each group 6 weeks after the operation (×40). B, Representative tartrate‐resistant acid phosphatase (TRAP)‐stained histologic distal femur sections from sham, ovariectomy (OVX), OVX + Trip and OVX + DMSO group (×400). C, Micro‐CT analysis of the distal femur from sham, OVX, OVX + Trip and OVX + DMSO group. D, Calculations of bone mineral density (BMD), trabecular number (Tb.N), bone surface area/total value (BS/TV), bone value/total value (BV/TV) and trabecular pattern factor (Tb.Pf). E, Serum IL‐6, TNF‐α, TRAcp5B and osteocalcin were examined (*P < .05, **P < .01, ***P < .001)

Animal experiments on OVX mice were carried out to simulate the clinical patients with complete hysterectomy and bilateral OVX. And the effects of triptolide on anti‐osteoporosis were explored. As H&E staining showed in Figure 6A, there was significant trabecular bone loss after OVX (*P* < .05). Triptolide significantly prevented the OVX‐induced trabecular bone loss (*P* < .01), however DMSO did not. As shown in Figure 6B, triptolide significantly decreased the OVX‐induced osteoclast increase. The results were confirmed by micro‐CT results, which includes the two‐dimensional structure and three‐dimensional structure and the BV/TV, BS/TV, Tb. N and BMD parameter statistics (Figure 6C‐H). Consistent with this, the serum levels of TRAcp5B, IL‐6 and TNF‐α induced by OVX were also reduced by M54 treatment (Figure 6I‐K). There is no significant influence on the serum levels of OCN (Figure 6L).

## 4 DISCUSSION

In this study, we investigated the effects of triptolide on osteoclastogenesis in vitro and on the OVX‐induced bone loss in vivo. The results showed that triptolide significantly inhibited RANKL/M‐CSF‐induced osteoclastogenesis through suppression PI3K‐AKT‐NFATc1 pathway, during which the expression of MDM2 was up‐regulated. Overexpression of NFATc1 and the treatment of AKT activator could reverse the triptolide effects. In vitro, the OVX‐induced bone loss of mice was significantly ameliorated by triptolide. This is the first study to demonstrate the effects of triptolide on OVX induced osteoporosis and bone metabolism. The results of this study also indicated that triptolide targeted to the upstream section of AKT to suppress PI3K‐AKT‐NFATc1 pathway and might regulate osteoclast cell cycle through AKT‐MDM2 pathway.

In human solid tumour, Bone loss (osteopenia) is one of the most common complications.^1^, ^2^ Surgical treatment is an effective therapy for the treatment of gynaecological tumour, such as cervical cancer, ovarian cancer and endometrial cancer.^3^, ^4^, ^5^ However, after complete hysterectomy and bilateral OVX, osteoporosis often occurs due to the oestrogen deficiency.^6^, ^7^ The specific mechanism of osteoporosis generation was still unclear; however, the excessive bone resorption induced by overactivated osteoclastogenesis was proved to be one of the most important factor.^26^ Thus, inhibiting osteoclast differentiation remains an important strategy for osteoporosis treatment.^27^ Except for osteoclastogenesis, inflammation activities also contribute large for the pathogenesis of osteoporosis.^28^ Oestrogen was proved to be a potent inflammation inhibitor.^29^, ^30^, ^31^ After surgical treatment of gynaecological tumour, the oestrogen drop rapidly and proinflammatory cytokines including TNF‐α and IL‐6 get elevated quickly and accelerate the bone loss.^32^, ^33^, ^34^, ^35^ These proinflammatory cytokines can stimulate osteoclast precursor cells to overexpress RANK, and activate osteoblast, mesenchymal stem cells and lymphocytes to express RANKL.^36^, ^37^

According to several recent studies, lots of anti‐osteoporosis drugs were derived from traditional Chinese medicine.^24^, ^38^, ^39^ Triptolide was derived from the traditional Chinese herb *lei gong teng*, which means thunder god vine.^19^ Triptolide was well known as a kind of anti‐cancer drugs,^40^ and recent study revealed that triptolide might have the effect to regulate bone metabolism.^41^, ^42^, ^43^, ^44^ Huang et al^43^ reported that triptolide could inhibit RANKL‐induced osteoclastogenesis and prevent osteolysis. Park^42^ reported that triptolide might inhibit RANKL or cancer cell‐induced osteogenesis. However, the effect of triptolide on bone‐loss disorders has not been studied. The mechanism how triptolide inhibited osteoclastogenesis and the role of PI3K‐Akt signalling also lack appropriated explanation. In this study, series of experiments were carried out to confirm the triptolide effect in bone metabolism and investigate the how triptolide regulate osteoclastogenesis.

In vivo, ovariectomized mice were used in this study to simulate the clinical patients with complete hysterectomy and bilateral OVX. H&E staining of the distal femur and micro‐CT indicated that triptolide decrease the bone loss, and TRAP staining indicated that triptolide inhibited osteoclastogenesis around the trabecular bone. At the same time, the level of proinflammatory cytokines including TNF‐α and IL‐6 was also ameliorated by triptolide.

Before formal experiments in vitro, CCK‐8 assay was carried out to investigate the cytotoxic effect of triptolide, of which the results (Figure 1E) indicated that triptolide (<25 nM) has no significant negative influence to cell proliferation, thus 3.125, 6.25, 12.5 and 25 Nm were selected for the following experiments. Following, the experiments showed that triptolide significantly prevented preosteoclast to differentiate to mature osteoclast. Bone resorption experiments indicated that triptolide also suppressed osteoclast function. Based on previous reports, the TRAP, Cathepsin K, TRAF6, CTR and MMP9 were selected as osteoclastogenesis‐related markers in this study.^22^, ^45^, ^46^ And we clarified that triptolide also inhibited the expression of MMP‐9, TRAP, C‐Src and Cathepsin K. These results confirmed the inhibiting effects of triptolide on osteoclastogenesis.

To investigate how triptolide inhibit osteoclastogenesis, Western blot assay was carried out to detect key protein and the phosphorylation of PI3K‐AKT‐NFATc1 pathway. Several studies well described the critical role of PI3K‐AKT pathway in differentiation and activation of osteoclast.^47^, ^48^, ^49^, ^50^ In brief, RANKL‐RANK interaction activates PI3K, which following phosphorylates AKT. The PI3K‐AKT signal pathway activation enhances NFATc1 expression and nuclei exportation, which promote osteoclast.^51^ In this study, the results of Western blot assay indicated that RANKL‐induced PI3K‐AKT pathway activation as well as NFATc1 expression was significantly ameliorated with triptolide treatment.

As PI3K‐AKT signal pathway suppressed, there was an increase of MDM2 expression. MDM2 is also the downstream of AKT. Activated AKT suppress MDM2 by phosphorylation, which results in the release p53 from negative control of phosphorylated MDM2.^52^, ^53^, ^54^ Following, activated p53 ultimately lead to progressive inflammation, premature atrophy and cell death.^52^, ^53^, ^54^, ^55^ The recent study has demonstrated that PI3K‐AKT inhibitor as well as MDM2 antagonist regulated tumour cells apoptosis, which showed significant anti‐cancer effect.^56^ However, the effect of PI3K‐AKT‐induced cell death has not been reported in osteoporosis. In this study, we demonstrated that the MDM2‐p53 interaction induced cell death contributed to the suppression of osteoclastogenesis.

In order to clarify how triptolide suppress PI3K‐AKT‐NFATc1 pathway, NFATc1 overexpression plasmid and AKT phosphorylation activator SC79 were used in the experiments in vitro.^57^ The results showed that the overexpression of NFATc1 and activation of AKT both ameliorated the effects of triptolide on osteoclastogenesis, which indicated that triptolide targeted to the upstream section of AKT.

According to previous researches, the anti‐cancer effects of triptolide was proved with cancer cells originated from different tissues, including breast, prostate and kidney.^19^ In our study, triptolide showed new biological effects of suppressing osteoclastogenesis, and protecting ovariectomized mice from bone loss. We hypothesize that the use of triptolide of cancer patients also prevents the common complication osteoclast‐mediated bone destruction.

Limitations within our study indicate the need for future work. Firstly, in our study, we demonstrated triptolide inhibit osteoclastogenesis through suppressing PI3K‐AKT‐NFATc1 pathway and clarified the target point located upstream of AKT. However, the exact mechanism how triptolide interact with the famous signal pathway still need to be defined. Secondly, we demonstrated the new effect of anti‐cancer drug triptolide to prevent osteoporosis and hypothesized the effect on cancer patients to prevent the complication of bone reconstruction. The hypothesis still needs to be confirmed with clinical trials.

In summary, this study proved that triptolide can suppress osteoclastogenesis in vitro, and prevent OVX‐induced bone loss in vivo. We demonstrated that the mechanism of triptolide effects is to inhibit PI3K‐AKT‐NFATc1 pathway by targeting to upstream section of AKT, and the MDM2‐p53‐induced cell death also contributed. In conclusion, the traditional Chinese herb derived anti‐cancer drug triptolide might be a promising therapy for the treatment of osteoporosis caused by tumour.

## CONFLICT OF INTEREST

The authors confirm that there are no conflicts of interest.

## AUTHOR CONTRIBUTION

CJ, LXQ and SJC designed this study. CJ, ZX and SYM finished the animal studies. CJ and LXQ finished BMMCs isolation. CJ, WSC and LXQ performed Western blotting. CJ and CX wrote the manuscript, and SJC reviewed the manuscript.

## Supporting information

FigureS1

## Data Availability

All data generated or analysed during this study are included in this article.
