# Supplementary figures and images for: Triptolide prevents bone loss via suppressing osteoclastogenesis through inhibiting PI3K‐AKT‐NFATc1 pathway
Source: J Cell Mol Med. 2020 Apr 28;24(11):6149–61. doi: 10.1111/jcmm.15229 (PMC7294126; doi:10.1111/jcmm.15229)

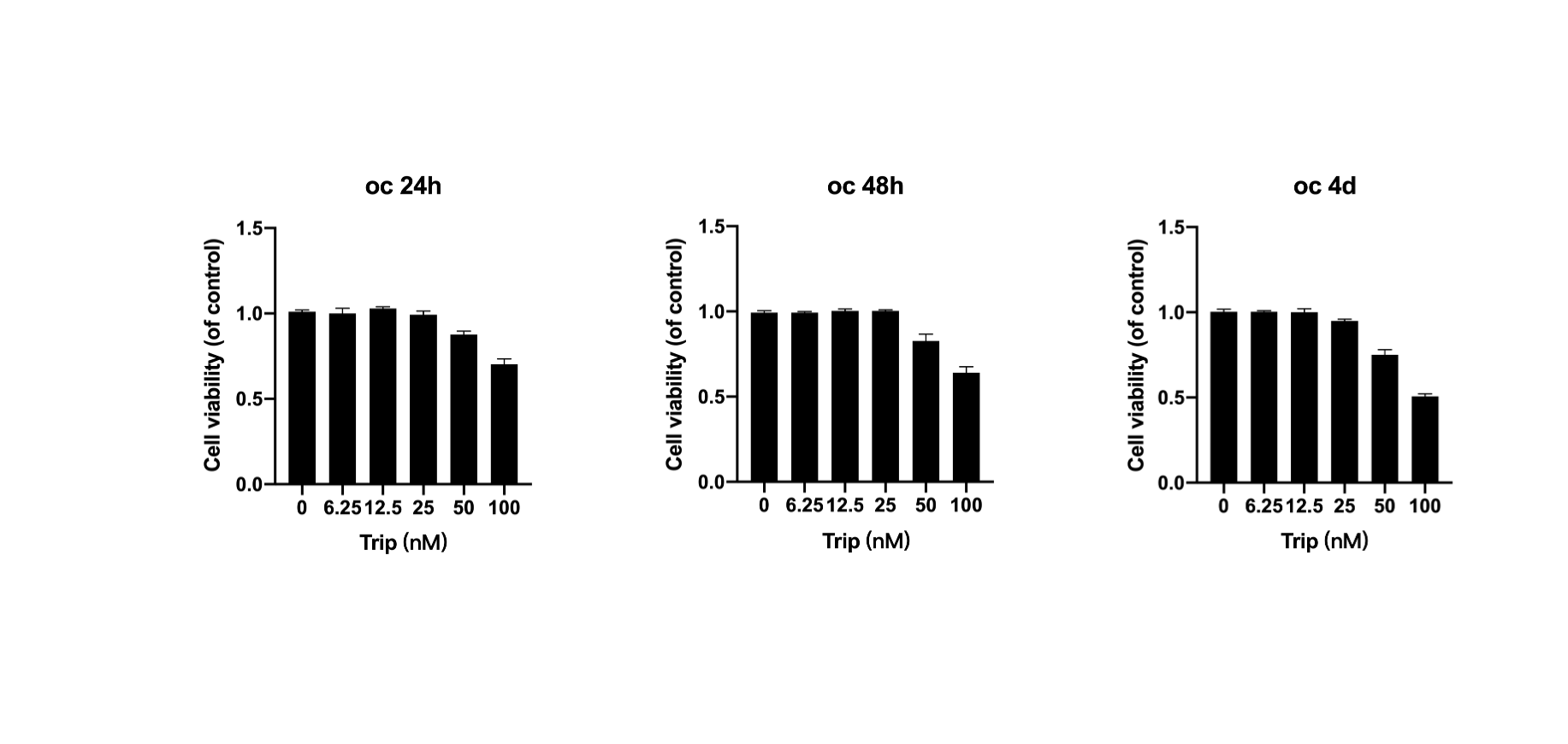

Supplement: Supplementary file 1 — FigureS1 [file JCMM-24-6149-s001.png]
